# Mixed adenocarcinoma, sarcomatoid carcinoma and adenosquamous carcinoma of the prostate: A case report

**DOI:** 10.3892/ol.2014.2493

**Published:** 2014-09-01

**Authors:** ZHONGFU ZHANG, YADONG WANG, QING ZHAO, GANHONG LI, XINGQI ZHAO, JUN LI, XIANXIN LI

**Affiliations:** 1 Department of Urological Surgery, Shenzhen Second People’s Hospital, The First Affiliated Hospital of Shenzhen University, Shenzhen, Guangdong 518036, P.R. China; 2 Department of Urology, Zunyi Medical College Fifth Affiliated Hospital, Zhuhai, Guangdong 519100, P.R. China; 3 Department of Urology, Peking University Shenzhen Hospital, Shenzhen, Guangdong 518036, P.R. China

**Keywords:** adenocarcinoma, sarcomatoid carcinoma, adenosquamous carcinoma, prostate carcinoma, p53

## Abstract

Adenosquamous carcinoma (ASC) and sarcomatoid carcinoma (SC) of the prostate are rare, but highly aggressive tumors. The occurrence of mixed carcinomas in the prostate is even more rarely reported. The present study reports the case of a 62-year-old male who was diagnosed with prostatic adenocarcinoma accompanied by multiple bone metastases, as shown by a needle biopsy and skeletal computed tomography scan. The patient was treated with hormonal therapy, but thereafter, specimens from a transurethral resection of the prostate (TURP) were found to be composed of three histologically distinct elements: ASC, SC and adenocarcinoma. The level of p53 was evaluated by immunohistochemistry in detail, and it was found that this was significantly increased in the TURP samples compared with the needle biopsy samples. The abnormal level of p53 was likely associated with the prognosis of the patient; the patient succumbed to prostate carcinoma two months after the confirmation of the diagnosis.

## Introduction

Prostate carcinoma is the most prevalent and lethal form of genitourinary cancer in males and is often identified following serum prostate-specific antigen (PSA) testing (1). Unusual histological prostatic carcinomas, which account for 5–10% of the carcinomas that originate in the prostate, have been described, including small cell carcinoma, sarcomatoid carcinoma (SC), squamous cell carcinoma and adenosquamous carcinoma (ASC) (2). Mixed prostate carcinomas are extremely rare and thus, no effective treatment has been identified and prognosis is poor. The present study reports the case of a prostate adenocarcinoma patient who developed mixed SC and ASC components following hormonal therapy. Written informed consent was obtained from the patient.

## Case report

On March 4, 2011, a 62-year-old male presented to the Department of Urology, Zunyi Medical College Fifth Affiliated Hospital (Guangdong, China), with recurrent symptoms of urinary retention and difficulty in voiding. During a routine physical examination, an incidental elevated PSA level of 30.5 ng/ml (normal range, 0–4 ng/ml) was identified. A bone scan revealed evidence of multi-focal bone metastases to certain regions, including the sternum, ribs, thoracic vertebrae, lumbar vertebrae, ilium, sacrum and femur. The patient underwent a prostate needle biopsy on March 10, 2011. Microscopic examination of the biopsy specimens revealed conventional poorly-differentiated adenocarcinoma of the prostate (Gleason score, 4+5). The patient was diagnosed with advanced prostate cancer, clinical stage T1cNxM1b, according to the Guidelines on Prostate cancer, European Association of Urology, 2008 (3).

**Figure 1:**
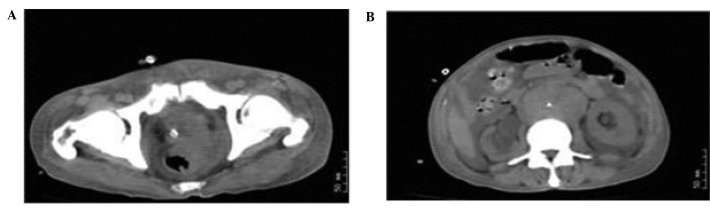
Computed tomography of the abdomen and pelvis. (A) The tumor infiltrated outside of the rectum. (B) The presence of hydronephrosis, retroperitoneal lymph node metastasis and seroperitoneum in the patient.

The patient was treated with adjuvant hormonal therapy consisting of bicalutamide (50 mg a day) and triptorelin (3.0 mg once every four weeks) for six months following the confirmation of the diagnosis, and the initial response was good. Treatment lasted until acute urinary retention occured. The serum PSA level decreased to 0.401 ng/ml after two months, then decreased further to 0.118 ng/ml after 3 months and finally decreased further to the lowest level of 0.082 ng/ml after 4 months. The patient was free of urinary symptoms 5 months after the prostate needle biopsy. Over the ensuing time, recurrent and progressive urinary symptoms developed and the patient was referred to the Department of Urology again due to acute urinary retention, on September 12, 2011. Catheterization was performed and the post-void residual urine volume was ~400 ml. The PSA level had risen to 15.630 ng/ml. A transurethral resection of the prostate (TURP) was performed to improve the urinary symptoms. Pathological analysis revealed a Gleason grade 5+5 adenocarcinoma of the prostate, with SC and ASC components. One month later, the PSA level increased rapidly to 122.591 ng/ml. Computed tomography of the abdomen and pelvis demonstrated multi-organ metastases, including metastases to the rectum and retroperitoneal lymph nodes, with a hydrothorax and ascites (Fig. 1). The patient’s clinical stage was T4N1M1c. The patient’s condition deteriorated rapidly and resulted in mortality due to multiple organ failure on November 26, 2011.

**Figure 2:**
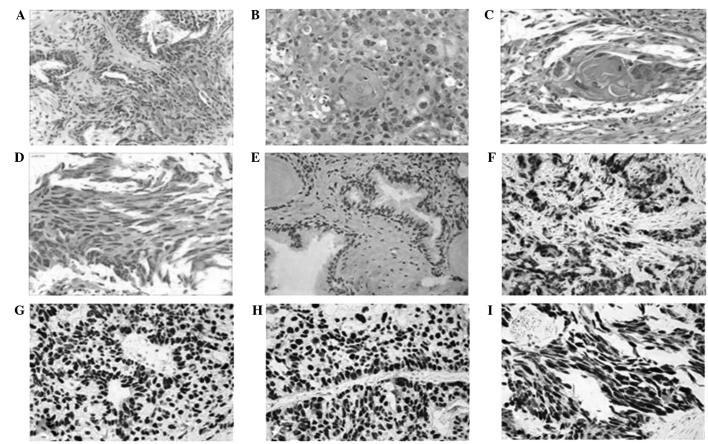
Hematoxylin and eosin and immunohistochemical staining of the prostatic specimen from the (A, E and F) needle biopsy and (B–D and G–I) transurethral resection of the prostate. (A) Prostatic adenocarcinoma. (B) Prostatic adenocarcinoma. (C) Squamous components; the centre of the image exhibits evidence of keratin pearl formation. (D) Sarcomatoid carcinoma (SC) sample exhibiting poorly-differentiated spindle cells with large hyperchromatic nuclei. (E) Gland area; negative for p53 expression. (F) Interstitial area; weakly positive for p53 expression. (G) Adenocarcinoma; positive for p53 expression. (H) Adenosquamous carcinoma; positive for p53 expression. (I) SC; positive for p53 expression. p53 expression is indicated by immunohistochemical staining for p53. (A, E and F) ×200 magnification; (B–D and G–I) ×400 magnification.

The first 13 biopsy specimens of the prostate revealed conventional poorly-differentiated adenocarcinoma with several acinar structures (Fig. 2A), with a Gleason score of nine out of 10, in four specimens from the left base and one specimen from the right midzone. Immunohistochemical studies revealed that the tumor cells were positive for PSA and CK, and negative for vimentin and the S-100 protein. The specimen from a TURP procedure 6-month after hormonal therapy, with a Gleason grade of 5+5, consisted of multiple fragments of necrotic tissue weighing ~15g. Microscopic examination of specimens from the TURP revealed different pathological features. Conventional prostatic adenocarcinoma were identified. The tumor was predominantly composed of sheets of small cells with round or oval-shaped nuclei, vesicular nuclear chromatin and sparse eosinophilic cytoplasm. Many of the cells appeared only as naked nuclei. Focal areas of necrosis were present. Immunohistochemical staining revealed that the tumor was positive for PSA and P504S. The final diagnoses of SC, ASC and adenocarcinoma were made based on histological studies (Fig. 2B–D). p53 was evaluated by immunohistochemistry (IHC) in detail and the results are shown in Fig. 2.

## Discussion

Mixed prostatic carcinoma is exceedingly rare and its pathogenesis is not clear (4). Previous studies have demonstrated that hormonal treatment and radiotherapy may lead to changes in the nature of the tumor (5). In the present study, the patient was diagnosed with adenocarcinoma, ASC and SC following hormonal treatment, and the patient’s condition progressively deteriorated despite active treatment.

The patient was diagnosed with mixed prostatic carcinoma by histological studies. The intercellular bridges and the keratin pearls that were found by hematoxylin and eosin (HE) staining confirmed the diagnosis of squamous cell carcinoma. In addition, HE staining confirmed the mesenchymal differentiation in the sarcomatoid component. In summary, the diagnoses of SC, adenocarcinoma and ASC were made based on pathological evidence.

Additionally, the p53 protein level in the biopsy samples was evaluated by IHC. To the best of our knowledge, IHC staining should be positive for wild-type p53 and for mutant-type p53, but the wild-type p53 is only barely detectable by IHC (6). The half-life of the wild-type p53 protein is short, but mutant p53 exhibits an altered structure that confers a longer half-life and greater stability compared with wild-type p53. As a result, the majority of positive cells represent mutant p53 (6). p53 is an important tumor suppressor that is involved in numerous molecular events, such as the regulation of DNA repair, apoptosis and senescence, and it is induced by various stress signals, including DNA damage and inflammation (7). However, previous studies have shown that the aberration of p53 may be the key event in the progression of carcinoma, as mutations in p53 result in a strong predisposition to cancer in humans (8). The majority of mutant p53 proteins lose their ability to bind wild-type p53-responsive elements and to regulate the expression of p53 transcriptional targets, thus losing tumor suppressor activity (9). Therefore, cellular preservation of mutated p53 may confer malignant potential, such as the capacity to metastasize, through gain of function activities (9). In the present study, the p53 level was markedly elevated in almost all the carcinoma cell nuclei in the TURP specimen compared with the needle biopsy staining, which indicated that p53 expression was negative in the gland area and weakly positive in the interstitial area (Fig. 2). The overexpression of p53 suggested the presence of mutations in p53. Previous studies have shown that mutations in p53 are possibly a mechanism to explain the change from castration-sensitive prostate carcinoma to castration-resistant prostate carcinoma (10). In the present patient, the change in carcinoma characteristics and the case prognosis was possibly associated with p53 mutation, according to the aforementioned studies.

In conclusion, mixed carcinoma in the prostate is a rare tumor with active clinical behavior. The disease often occurs following radiation or hormonal therapy, but the mechanism underlying its development is unclear. The present study speculates that the variation in carcinoma type is possibly associated with mutations in p53.
